# Response to Gaseous NO_2_ Air Pollutant of *P. fluorescens* Airborne Strain MFAF76a and Clinical Strain MFN1032

**DOI:** 10.3389/fmicb.2016.00379

**Published:** 2016-03-31

**Authors:** Tatiana Kondakova, Chloé Catovic, Magalie Barreau, Michael Nusser, Gerald Brenner-Weiss, Sylvie Chevalier, Frédéric Dionnet, Nicole Orange, Cécile Duclairoir Poc

**Affiliations:** ^1^Laboratory of Microbiology Signals and Microenvironment EA 4312, Normandy University, University of Rouen, SéSa, IRIBEvreux, France; ^2^Aerothermic and Internal Combustion Engine Technological Research CentreSaint Etienne du Rouvray, France; ^3^Institute of Functional Interfaces, Karlsruhe Institute of TechnologyKarlsruhe, Germany

**Keywords:** airborne, *Pseudomonas fluorescens*, nitrogen dioxide, biofilm, antibiotic sensitivity, motility, air pollution

## Abstract

Human exposure to nitrogen dioxide (NO_2_), an air pollutant of increasing interest in biology, results in several toxic effects to human health and also to the air microbiota. The aim of this study was to investigate the bacterial response to gaseous NO_2_. Two *Pseudomonas fluorescens* strains, namely the airborne strain MFAF76a and the clinical strain MFN1032 were exposed to 0.1, 5, or 45 ppm concentrations of NO_2_, and their effects on bacteria were evaluated in terms of motility, biofilm formation, antibiotic resistance, as well as expression of several chosen target genes. While 0.1 and 5 ppm of NO_2_did not lead to any detectable modification in the studied phenotypes of the two bacteria, several alterations were observed when the bacteria were exposed to 45 ppm of gaseous NO_2_. We thus chose to focus on this high concentration. NO_2_-exposed *P. fluorescens* strains showed reduced swimming motility, and decreased swarming in case of the strain MFN1032. Biofilm formed by NO_2_-treated airborne strain MFAF76a showed increased maximum thickness compared to non-treated cells, while NO_2_ had no apparent effect on the clinical MFN1032 biofilm structure. It is well known that biofilm and motility are inversely regulated by intracellular c-di-GMP level. The c-di-GMP level was however not affected in response to NO_2_ treatment. Finally, NO_2_-exposed *P. fluorescens* strains were found to be more resistant to ciprofloxacin and chloramphenicol. Accordingly, the resistance nodulation cell division (RND) MexEF-OprN efflux pump encoding genes were highly upregulated in the two *P. fluorescens* strains. Noticeably, similar phenotypes had been previously observed following a NO treatment. Interestingly, an *hmp*-homolog gene in *P. fluorescens* strains MFAF76a and MFN1032 encodes a NO dioxygenase that is involved in NO detoxification into nitrites. Its expression was upregulated in response to NO_2_, suggesting a possible common pathway between NO and NO_2_ detoxification. Taken together, our study provides evidences for the bacterial response to NO_2_ toxicity.

## Introduction

Most world-wide cities have serious air-quality problems, which have attracted attention in the past decade. One of the most common source of air pollution is engine emissions, which include, among other toxic molecules, the nitrogen oxides (NO_x_; reviewed in Sher, 1998; Skalska et al., 2010). The general term NO_x_ includes nitric oxide (NO) and nitrogen dioxide (NO_2_). NO in turn is able to damage bacterial cells interacting with bacterial proteins (McLean et al., 2010; Laver et al., 2013) and DNA (Tamir et al., 1996; Burney et al., 1999) either directly, or via formation of reactive nitrogen species (RNS), causing alterations in bacterial metabolism, among which respiration, and homeostasis. As a result, bacteria have developed specific NO detoxification pathways and defense mechanisms (Cruz-Ramos et al., 2002; Flatley et al., 2005; Spiro, 2007). In order to counteract the NO-mediated respiratory arrest (Husain et al., 2008), the detoxification processes are completed in several bacteria by metabolism reprogramming (Auger et al., 2011; Auger and Appanna, 2015). NO was furthermore identified as a signaling molecule, which promotes the biofilm dispersion in various bacterial strains, including *Pseudomonas aeruginosa* (Barraud et al., 2009; Cutruzzola and Frankenberg-Dinkel, 2015) and *P. putida* (Liu et al., 2012). This molecule is also known to modulate bacterial antibiotic sensitivity, protecting bacteria from a wide range of antibacterial agents (Gusarov et al., 2009; McCollister et al., 2011; van Sorge et al., 2013), such as vancomycin and daptomycin (van Sorge et al., 2013). Contrary to NO, NO_2_ has a low solubility in water (Augusto et al., 2002). Thence NO_2_ in aqueous media concerned a few reports in the microbiological context. However, in natural environments NO is unstable and quickly oxidized to form NO_2_ (Skalska et al., 2010), considered as a major air pollutant. Its atmospheric level is ruled by European environmental commission and World Health Organization (INERIS, 2011; Reduction of pollutant emissions from light vehicles, 2015; WHO |Ambient (outdoor) air quality health, 2015). NO_2_ toxicity to human health is well documented and is known to increase cardiovascular diseases (Chaloulakou et al., 2008), or to aggravate respiratory symptoms especially in children (Pershagen et al., 1995; Chauhan et al., 1998). On the opposite, the stress promoted by NO_2_ was poorly evaluated on bacteria.

It is increasingly evident that the air is a biotic environment, containing bacteria as one of the major compounds of primary atmosphere aerosol particles (Burrows et al., 2009b; Després et al., 2012). Mean airborne bacterial concentrations can indeed be greater than 1 10^4^ cells m^−3^ (Bauer et al., 2002; Burrows et al., 2009a). Although unstable, the air microbiota is frequently constituted with members of *Pseudomonas* genus (Fang et al., 2007; Pearce et al., 2010; Després et al., 2012; Dybwad et al., 2012; Šantl-Temkiv et al., 2015). Among these highly versatile elements, the *P. fluorescens* strains are widely adaptable and distributed (Bodilis et al., 2004) in all major natural environments, including water (Bodilis et al., 2004), soil (Varivarn et al., 2013) and clouds (Ahern et al., 2007). Several *P. fluorescens* strains were also found to promote humans acute infections and were reported in clinical samples of immuno-compromised patients (Chapalain et al., 2008; Scales et al., 2014). All these properties make *P. fluorescens* a good model for further investigations of airborne bacteria.

We have investigated in previous studies the microbiota (bacteria, yeasts and fungi) of Rouen harbor terminal (France) (Morin et al., 2013). Thus, several *P. fluorescens* strains were isolated. Among them, the airborne *P. fluorescens* strain MFAF76a was characterized as a virulent strain, particularly its exoproducts against human epithelial pulmonary cells (Duclairoir Poc et al., 2014). The aim of this study is to investigate the physiological response of airborne *P. fluorescens* MFAF76a to NO_2_ as a marker of air pollution in terms of motility, biofilm formation and antibiotic resistance. This response was compared to that of the clinical strain *P. fluorescens* MFN1032 isolated from the sputum of a pneumonia-suffering patient (Chapalain et al., 2008). The parameters of bacterial NO_2_ exposure were adapted to mimic real-life air conditions. Thus, the two strains were exposed to gaseous NO_2_ at three concentrations: 0.1 ppm as an annual guideline value (WHO |Air quality guidelines - global update, 2005) 5 ppm as the threshold causing reversible effects on human health, and 45 ppm as a high NO_2_ concentration provoking irreversible effects (INERIS, 2011).

## Material and methods

### Strains and growth conditions

Cyan Fluorescent Protein (CFP)-labeled *P. fluorescens* MFN1032 and MFAF76a were used in this study. The strains and plasmids are listed in Table S1. The 729-bp *cfpopt* gene, encoding the CFP, was extracted from pTetONCFPopt plasmid (Sastalla et al., 2009) using PstI and Xmal enzymes (NEB, Ipswich, USA). Then CFP cassette was separated by 1% agarose gel electrophoresis and purified with QIAquick Gel Extraction Kit (Qiagen, Hilden, Allemagne). The pPSV35 vector (Rietsch et al., 2005) was digested using PstI and Xmal and purified using QIAquick PCR Purification Kit (Qiagen, Hilden, Allemagne). The CFP cassette was then cloned into the PstI and Xmal sites of the pPSV35 vector. The resulting pCFP vector was introduced into One Shot® TOP10 Chemically Competent *E. coli* (LMSM collection) by heat shock. After antibiotic selection of the clones (gentamycin 15 μg/mL), the transformation was confirmed by confocal laser scanning microscope (CLSM 710, ZEISS). The obtained plasmid was then extracted from *E. coli* using QIAprep Spin Miniprep Kit (Qiagen, Hilden, Allemagne) and introduced into *P. fluorescen*s strains by electroporation. The transformants were selected in LB containing 15 μg/mL of gentamycin and fluorescence was assayed using CLSM.

**Figure 1:**
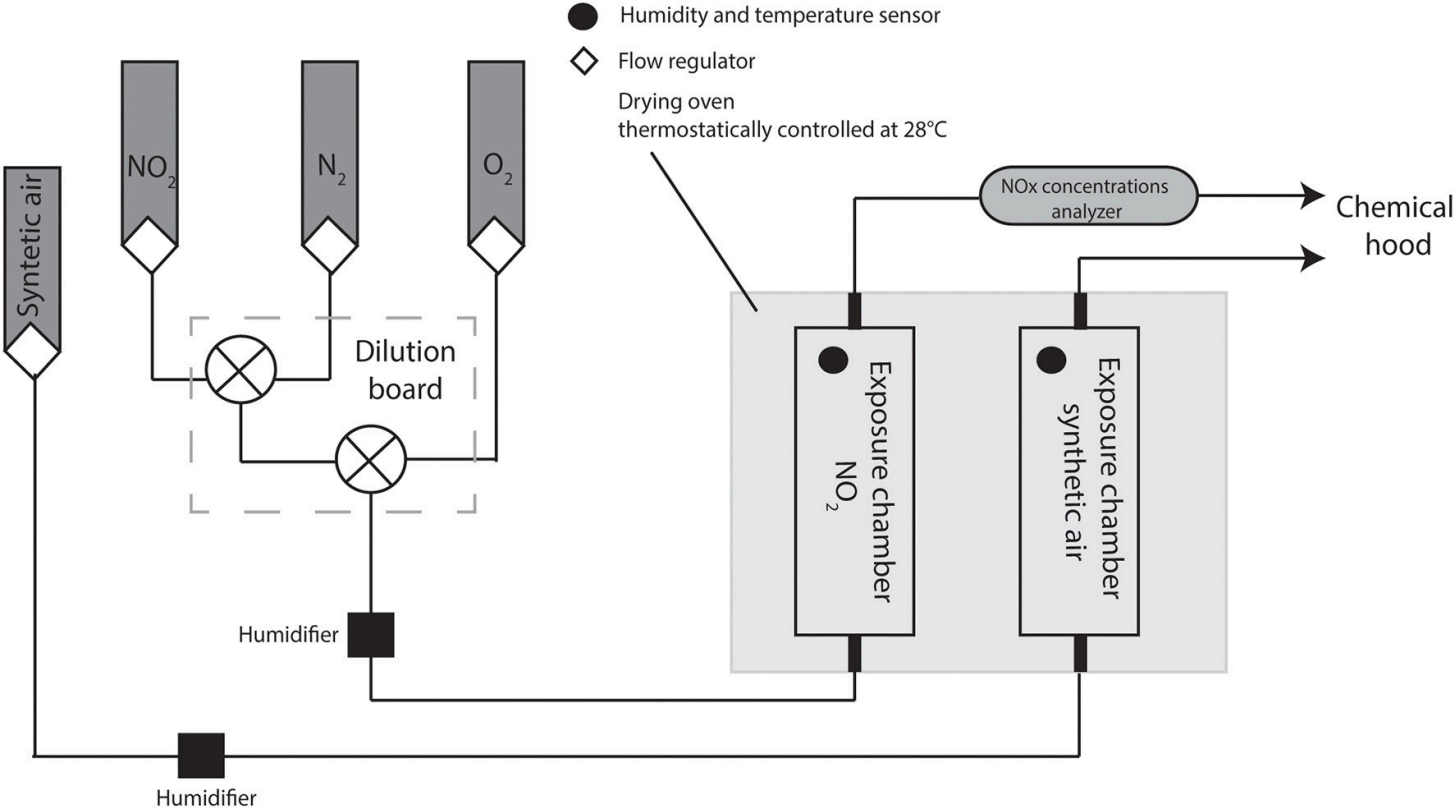
**Schematic representation of NO_2_ gas delivery system**. Bacterial NO_2_ exposure was done in gas phase for 2 h. Two exposure chambers (one for the NO_2_ exposure, the second one for the control—synthetic air exposure) were used. The gases, including NO_2_, N_2_ and O_2_ were mixed together to obtain pre-calculated concentrations of NO_2_ and maintain the O_2_/N_2_ ratio at 2/8 (v/v). NO_2_ concentrations, temperature and relative humidity were controlled.

Bacteria were grown at 28°C under limited agitation (180 rpm) in DMB (Davis Medium Broth) minimal medium with 2.16 g/L glucose as carbon source (Duclairoir-Poc et al., 2011). Overnight cultures were diluted (A_580_ = 0.08) in fresh DMB and grown to the end of exponential phase (A_580_ = 2, 13 × 10^8^ CFU/mL). Bacterial cultures at the end of exponential growth phase (about 3 × 10^7^ bacteria per filter) were transferred on cellulose nitrate membrane filter (0.45 μm, pore size 0.2 μm, diameter 47 mm, Sartorius Biolab Products, Gottingen, Germany) and grown on DMB agar plates at 28°C for 4 h to obtain a single layer's bacterial population. After 4 h of incubation, the cellulose membranes containing bacteria were placed on agar one-well dishes (size 127.8 × 85.5 mm, Thermo Scientific Nunc, Roshester, USA), which were directly transferred into the gas delivery device (Figure 1).

### Exposition to nitrogen dioxide

In order to mimic the atmospheric conditions, bacterial NO_2_ exposure was achieved in gas phase for 2 h, according to Ghaffari et al. (2005). The gas delivery device consisted of two sterile cylindrical Plexiglas exposure chambers (one for the NO_2_ exposure, the second one for the control—exposure to synthetic air). The exposure chambers were deposed in a drying oven at 28°C (Figure 1). The NO_2_, N_2_, and O_2_ obtained from Air Liquide GMP Europe (Mitry-Mory, France) were mixed together using digital mass flow regulators (Alicat Scientific, Inc., Tucson, USA) in order to get pre-calculated concentrations of NO_2_ and maintain the O_2_/N_2_ ratio at 2/8 (v/v). The resulting gas mixture and the synthetic air were routed independently to each of the exposure chamber at a constant flow rate of 2 L/min, allowing parallel treatment of bacteria originating from the same bacterial culture. After passing through the exposure chamber, the NO_2_ concentrations were monitored by AC32M nitrogen oxides analyzer (Environnement S.A, Poissy, France) and safely vented to a chemical hood. Temperature and relative humidity data were monitored to control reliable steady-state environmental conditions inside the exposure chambers. Three concentrations of NO_2_ (0.1 ppm; 5 ppm and 45 ppm) were used in this study. After exposure, bacteria were diluted to A_580_ = 2 in sterile saline solution and used for the subsequent experiments.

### Antibiotic sensitivity assays

After NO_2_ exposure, bacterial sensitivity to ciprofloxacin, chloramphenicol, tobramycin and kanamycin (Sigma-Aldrich, St. Quentin Fallavier, France) was assayed. The minimum inhibitory concentration (MIC) was determined by the broth microdilution method achieved in DMB. Briefly, NO_2_-exposed bacteria were diluted to A_580_= 0.08 and added to a 96-well test plate (Nunc™, Roskilde, Denmark) containing different concentrations of antibiotics in triplicate. The test plates were incubated at 28°C for 24 h. Synthetic air- exposed bacteria were used as control. MIC was defined as the lowest antibiotic concentration that inhibited bacteria growth as determined by turbidimetry at A_580_.

Growth inhibition assays were achieved as previously described (van Sorge et al., 2013). Exposed bacteria were diluted in DMB supplemented by the indicated antibiotics in subinhibitory concentrations (the last antibiotic concentrations allowing bacterial growth). Bacteria were added to Bioscreen Honeycomb plates (Oy Growth Curves Ab Ltd., Helsinki, Finland) in a total volume of 200 μL of DMB (A_580_ = 0.08). Growth was measured every 15 min (A_580_) for 24 h. The NO_2_ effect on the bacterial antibiotic sensitivity was calculated as the percentage of bacterial growth with antibiotics after NO_2_ exposure on the bacterial growth with antibiotics after exposure to synthetic air, using the following formula: 100 × A_580_ NO_2_ exposed bacteria/A_580_ synthetic air exposed bacteria (%).

### Motility assays

Swimming and swarming motility assays were performed on agar plates using DMB containing 0.2% (wt/vol) and 0.5% (wt/vol) agar, respectively, as previously described (Déziel et al., 2001). Briefly, 5 μL of NO_2_ or synthetic air- exposed bacteria were spotted on the surface of agar plates. The resultant diameters of swim and swarm zones were measured after 24 h of incubation at 28°C. Motilities were assayed in three independent experiments with three replicates for each experimental condition.

### Biofilm monitoring by confocal laser scanning microscopy

NO_2_ or synthetic air- exposed bacteria were diluted in sterile saline solution to A_580_= 1 to avoid bacterial multiplication, and added to glass-bottom dishes (SensoPlate™, VWR, Fontenay-sous-Bois, France). After 2 h of incubation at 28°C, planktonic bacteria were removed and bacterial adhesion on glass-bottom dishes was observed using a confocal laser scanning microscope (CLSM 710, ZEISS) with an immersion objective 63 ×. After addition of DMB, the samples were incubated at 28°C for 24 h. Biofilms were rinsed with saline solution and observed using CLSM. All biofilm assays were performed in three independent experiments with two replicates for each experimental condition. The biofilm thickness and related biomass (bacterial volume, μm^3^/μm^2^) were estimated from 6 fields on 3 independent experiments using COMSTAT software (Heydorn et al., 2000).

### Gene sequences identification

The non-annotated genome drafts of MFN1032 and MFAF76a were used to identify the corresponding nucleotide sequences (data not shown). Homologous sequences search in *P. fluorescens* annotated genomes was performed using pseudomonas genome database (http://pseudomonas.com/). The conserved nucleotide sequences were identified in *P. fluorescens* MFN1032 and MFAF76a using Blast+ (Stand-alone) software (v. 2.2.30, NCBI) according to Altschul et al. (1997), and are listed in Table S2.

### Extraction and quantification of bis-(3′, 5′)-cyclic dimeric guanosine monophosphate (c-di-GMP)

Extraction and quantification of intracellular c-di-GMP level were performed in NO_2_ or synthetic air- exposed bacteria as previously described (Spangler et al., 2010; Strehmel et al., 2015). Identification and quantification of c-di-GMP was performed using three specific mass transitions from molecule ion m/z 691 to the product ions: m/z 152, m/z 135, and m/z 540. The external calibration was carried out at c-di-GMP concentrations ranging from 10 ng to 200 ng in 500 μL H_2_O using the internal standard cXMP (50 ng). The resulting concentrations of c-di-GMP were normalized against total protein contents of respective cultures, which was determined by the bicinchoninic acid assay (Smith et al., 1985). All experiments were performed in three replicates for each experimental condition.

### Quantitative RT-PCR

Total RNA was prepared by the hot acid-phenol method (Bouffartigues et al., 2012) from NO_2_-exposed and not bacteria. Residual DNAs were eliminated by acid phenol treatment. The absence of DNA was confirmed by showing that PCR reactions failed without prior cDNA synthesis. RNAs were nonspecifically converted to single stranded cDNAs using the High Capacity cDNA Archive Kit (Applied Biosystems). Synthesis of cDNAs and real time PCR, allowing the quantification of mRNAs of interest were performed as previously described (Gicquel et al., 2013) using primers listed in Table S3.

### Statistical analysis

All experiments were carried out several times. To assess the significance of differences between the obtained data, Mann-Whitney test or pairwise strain comparisons (*t*-test) were applied and quantified the significance as (^*^) for *p* < 0.05, (^**^) for *p* < 0.01 and (^***^) for *p* < 0.001.

## Results and discussion

NO_2_ is one of the most common air pollutants, but its effects on the air microbiota is poorly studied. In order to assess the bacterial response to NO_2_, airborne *P. fluorescens* MFAF76a and clinical control MFN1032 strains were exposed to gaseous NO_2_ (as shown Figure 1) at 0.1, 5, or 45 ppm concentrations, and their effects on bacteria were evaluated in terms of motility, biofilm formation, antibiotic resistance, as well as expression of several chosen target genes. While 0.1 and 5 ppm of NO_2_ did not lead to any significant modification of the studied parameters in both the bacteria (data not shown), several alterations were observed when the bacteria were exposed to 45 ppm of gaseous NO_2_. We thus chose to focus on this concentration.

### No_2_-mediated modifications of bacterial biofilm

**Figure 2:**
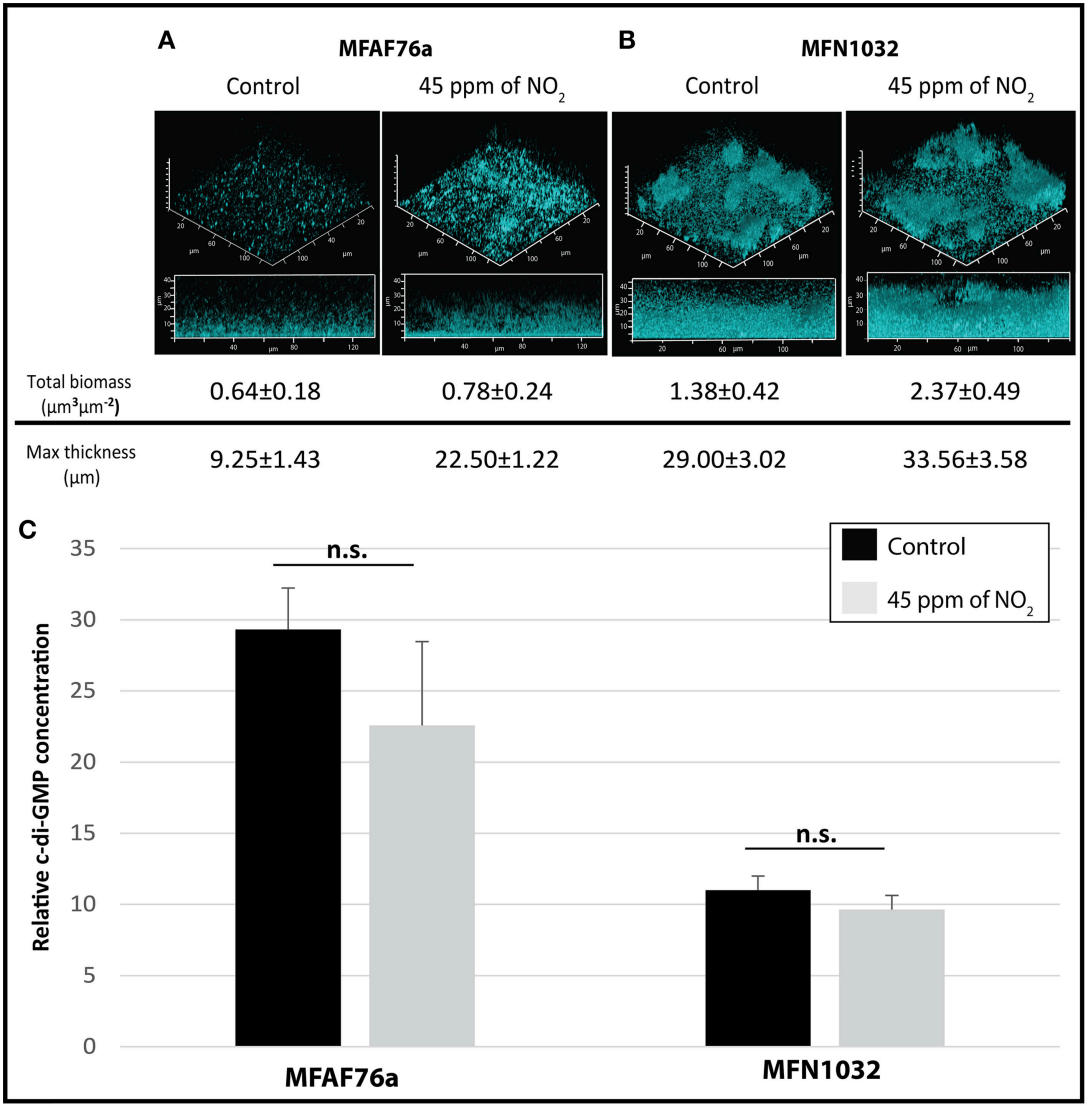
**NO_2_ effect on *P. fluorescens* biofilm and intracellular c-di-GMP level. (A)** Airborne MFAF76a and **(B)** clinical MFN1032 *P. fluorescens* strains were exposed in triplicate to 45 ppm of NO_2_. Biofilm formation was analyzed in static conditions after 24 h development using confocal laser scanning microscope. The biofilm biomass and the maximum thickness were estimated from 6 fields on 3 independent experiments using COMSTAT software. Intracellular c-di-GMP concentrations **(C)** were measured in triplicate by LC-MS/MS for control (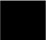) and 45 ppm of NO_2_ treated (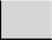) MFAF76a and MFN1032. Obtained results are presented as average values ± SEM. Statistical significance was calculated by the non-parametric Mann-Whitney-Test. n.s., non-significant.

In order to test the NO_2_ effect on *P. fluorescens* biofilm, both airborne MFAF76a and clinical MFN1032 were exposed to gaseous NO_2_ and synthetic air and grown for 4 h in static conditions. In the control condition, the airborne strain MFAF76a produced only a poorly structured biofilm with low biomass and thickness (Figure 2A). To the best of the authors' knowledge this is the first time that the biofilm of airborne *P. fluorescens* strain was investigated. On the opposite, the clinical strain MFN1032 was able to form a structured mushroom-like biofilm, with about 2 and 3 fold more biomass and thickness than the airborne strain MFAF76a, respectively (Figure 2B, control). These data are consistent with previous studies showing that clinical strains can strongly adhere and form structured biofilms (Rossignol et al., 2008; Ma et al., 2009). After NO_2_ exposition,the airborne strain MFAF76a produced biofilms with about 3 fold increase of the maximal thickness, while the biomass was similar (Figure 2A), when compared to synthetic air treatment. These data suggest that NO_2_ led to induce biofilm formation in this strain. Accordingly, similar NO concentrations were previously found to promote an increase of biofilm formation in *P. aeruginosa* (Barraud et al., 2006), suggesting a common effect between NO and NO_2_ treatment. On the other hand, NO_2_ exposure of the clinical strain MFN1032 led to a 1.7 fold increase in biofilm production in terms of biomass, while the maximal thickness was unchanged (Figure 2B). Taken together these data suggest that the NO_2_-mediated biofilm modifications are strain-dependent. Since we have shown previously that airborne MFAF76a expresses a virulence activity toward A549 epithelial pulmonary cells (Duclairoir Poc et al., 2014), these data suggest that elevated concentrations of NO_2_ increases biofilm formation in potentially virulent airborne strain and may represent a sanitary risk.

Since biofilm formation is related to increased c-di-GMP production (Ha and O'Toole, 2015), we next quantified the c-di-GMP levels after NO_2_ or synthetic air exposure. As shown in Figure 2C, both NO_2_-exposed *P. fluorescens* strains did not exhibit statistically significant variations of intracellular c-di-GMP concentrations. This was quite surprising since observed in our study NO_2_ mediated biofilm induction. NO-mediated reduction of the intracellular c-di-GMP level leading to dispersion of *P. aeruginosa* biofilms has been related to increase phosphodiesterases (PDEs) activity, and as a consequence to promote the switch between the biofilm and the planktonic ways of life (Petrova and Sauer, 2012; Roy et al., 2012; Li et al., 2013; Petrova et al., 2014). In this bacterium, the following PDEs, including DipA, MucR, NdbA and BdlA, are enzymes that are involved in c-di-GMP catabolism (Petrova and Sauer, 2012; Roy et al., 2012). The mRNA levels of *dipA, mucR, ndbA* and *bdlA* genes (KT186437, KT186445, KT186444 and KT186436 respectively, Table S2) were quantified by qRT-PCR experiments, in the two strains, that were both previously exposed to NO_2_ or synthetic air. For the two strains, NO_2_ exposure did not lead to any modification in gene expression (data not shown). Altogether, these data suggest that (i) NO_2_ may have an effect on the structure or on the biomass of the biofilm, in case of the studied airborne or clinical strains, respectively, (ii) these phenotypes would not be related to variations of the intracellular c-di-GMP levels, and (iii) NO and gaseous NO_2_ may have a common and concentration-dependent effect on biofilm formation.

### No_2_ reduced bacterial motility

Since biofilm structure and production were not implemented by c-di-GMP level in our conditions, we next assayed the effects of NO_2_ on bacterial motility, since appendices like flagella and type IV pili are also involved in the first step of biofilm formation, i.e., adhesion (Caiazza et al., 2007; Guttenplan and Kearns, 2013).

**Figure 3:**
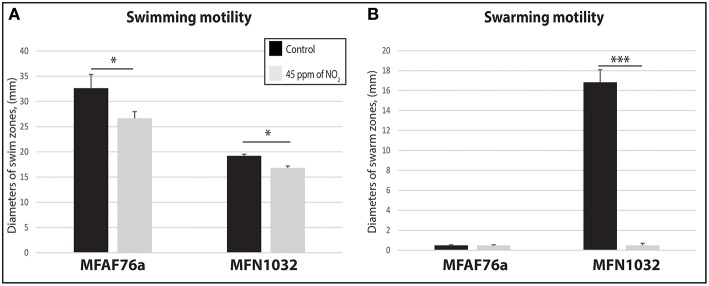
**NO_2_ decreases *P. fluorescens* motility**. Airborne MFAF76a and clinical MFN1032 *P. fluorescens* strains were exposed in triplicate to 45 ppm of NO_2_(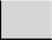). Swimming **(A)** and swarming **(B)** motilities were assayed on DMB-swim/swarm plates after 24 h incubation. The motile bacterial movement was evaluated in three independent experiments with three replicates. The data were compared with control exposed to synthetic air (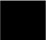). Obtained results are presented as average values ± SEM. Statistical significance was calculated by the non-parametric Mann-Whitney-Test *p* < 0.05 (^*^) and < 0.001 (^***^).

Swimming concerns motility in a liquid medium, mediated by production and activity of flagella. As shown on Figure 3A, gaseous NO_2_ exposition significantly decreased the swimming motility of both strains, suggesting an impairment of the flagellum production and/or activity.

Swarming is a complex motility that has been related to functional flagella, type IV pili and production of biosurfactants like cyclolipopeptides (Duclairoir-Poc et al., 2011) for some *P. fluorescens* strains, or rhamnolipids for *P. aeruginosa* strains (Caiazza et al., 2005). The airborne strain MFAF76a was unable to swarm in tested experimental conditions. On the opposite, MFN1032 is a swarmer clinical strain (Rossignol et al., 2008). As shown in Figure 3B, exposition to NO_2_ but not to synthetic air led to fully inhibit the swarming motility of this strain.

Taken together, our data show that gaseous NO_2_ treatment results in a decreased motility in both of the studied strains. This decrease in motility could be a consequence of a lower production of the required appendices. Alternatively it could also be due to lower appendices activity, suggesting that they could increase the attachment of the bacterium on the glass slide. This phenotype would then be consistent with the increase in biofilm maximal thickness in case of the airborne strain, and biomass in case of the clinical strain. However, to date, the switch between motility and biofilm had frequently been associated to variations in the c-di-GMP level (Ha and O'Toole, 2015), but, herein, the gaseous NO_2_-mediated differences in terms of biofilm structure could not be related to any c-di-GMP level variations.

### Effect of no_2_ on mexef-oprn efflux pump expression and antibiotic resistance

To further characterize the effects of gaseous NO_2_ on bacterial physiology, we next assayed antibiotic resistance. Since NO, a member of RNS, was found to induce the expression of *mexEF-oprN* genes (Fetar et al., 2011) and modulate bacterial resistance to fluoroquinolones, chloramphenicol and aminoglycosides (Gusarov et al., 2009; McCollister et al., 2011; van Sorge et al., 2013), we investigated the effect of gaseous NO_2_ on these phenotypes.

**Figure 4:**
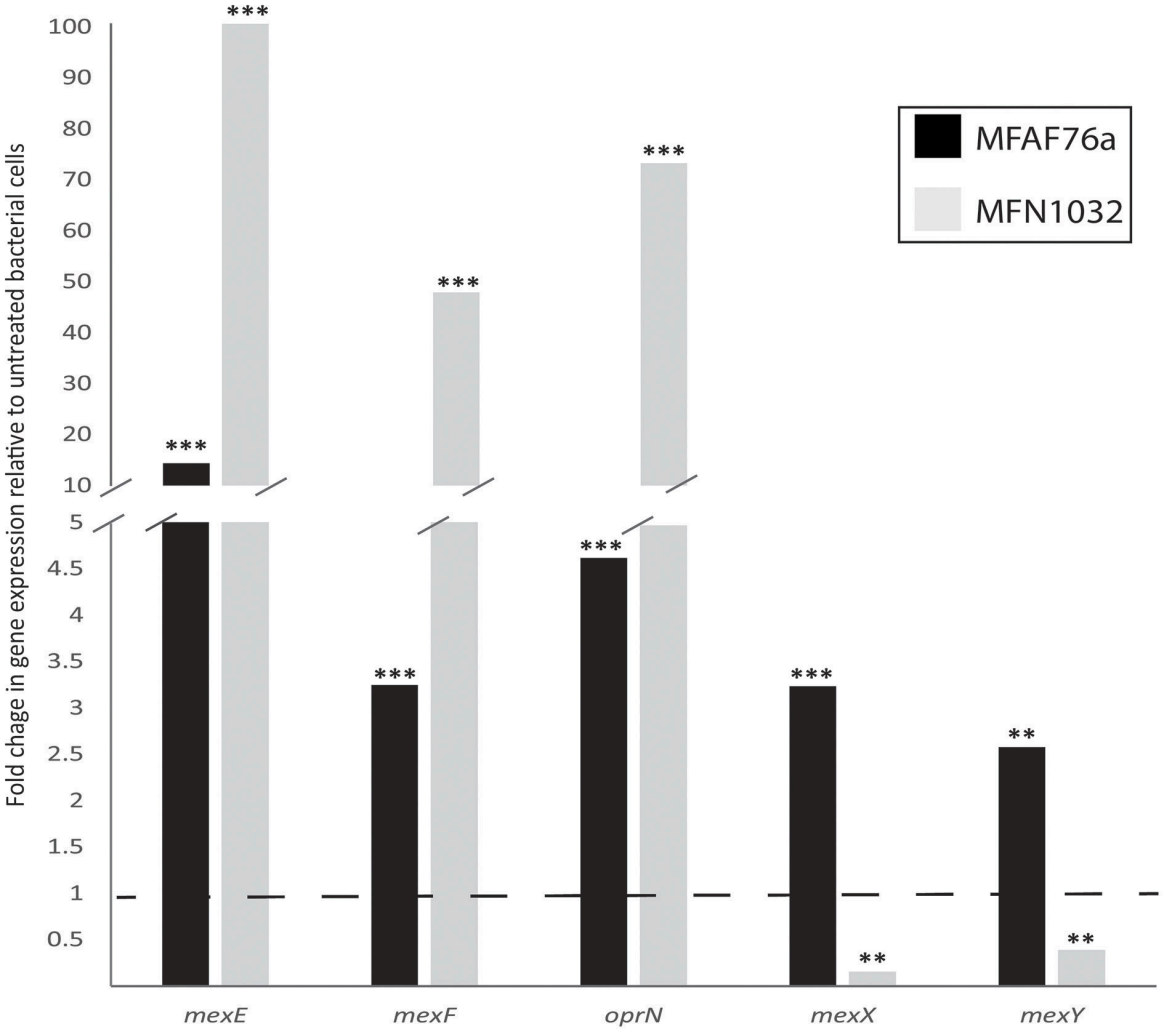
**NO_2_ effect on MexEF-OprN and MexXY efflux pump gene transcription**. The nucleotide sequences of the *mexEF-, oprN*- and *mexXY*-homolog genes were obtained using the non-annotated genome drafts of airborne MFAF76a (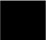) and clinical MFN1032 (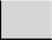) *P. fluorescens*. The GenBank accession numbers of nucleotide sequences are listed in Table S2. Quantification of mRNA level was assayed using qRT-PCR on RNAs extracted from NO_2_- and synthetic air- exposed *P. fluorescens*. The PCR reactions were performed in triplicate and the standard deviations were lower than 0.15 Ct. Statistical analysis used pairwise strain comparisons (*t*-test) *p* < 0.01 (^**^) and < 0.001 (^***^). Dotted line shows the gene expression in synthetic air- exposed control.

**Figure 5:**
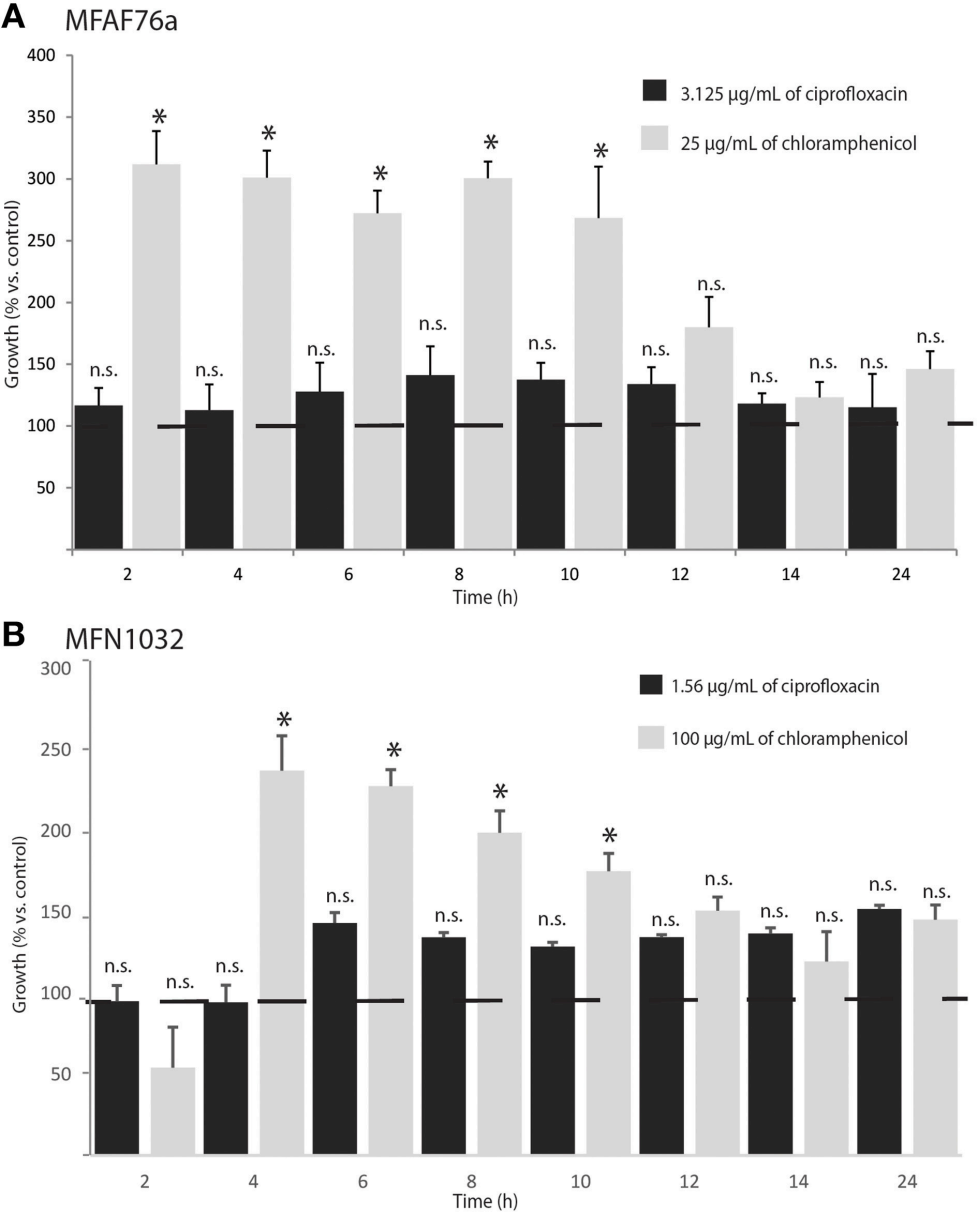
**NO_**2**_ protects ***Pseudomonas fluorescens*** from chloramphenicol toxicity**. After 2 h exposure to 45 ppm of NO_2_, growth of airborne MFAF76a **(A)** and clinical MFN1032 **(B)** *P. fluorescens* with ciprofloxacin (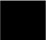) and chloramphenicol (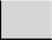) was assayed. Growth curves were performed with ciprofloxacin (3.125 μg/mL for MFAF76a and 1.156 μg/mL for MFN1032) and chloramphenicol (25 and 100 μg/mL respectively), and A_580_ was recorded at the indicated time points. The control sample was bacteria exposed to synthetic air, and grown in presence of antibiotics in indicated concentrations. The data are shown as percentages of growth relative to synthetic air-exposed control. Pooled data from three independent experiments in duplicate ± SEM are reported. Statistical significance was calculated by the non-parametric Mann-Whitney-Test *p* < 0.05 (^*^); n.s., non-significant. Dotted line shows the control (100%).

In order to study the effect of NO_2_ on MexEF-OprN efflux pump, the transcription levels of *mexE, mexF* and *oprN* genes (KT070324, KT070321 and KT070325 for MFAF76a; KT070323, KT070322 and KT186432 for MFN1032, respectively) were compared using qRT-PCR in two *P. fluorescens* strains exposed or not to 45 ppm of NO_2_. In airborne and clinical strains, the *mexE* mRNA level was increased by almost 14- and 100-fold respectively; that of *mexF* almost 3.5- and 47-fold respectively and that of *oprN* almost 4.6- and 73-fold respectively (Figure 4). These data show that NO_2_ promoted *mexEF-oprN* expression, potentially causing modifications in *P. fluorescens* antibiotic resistance. We next tested the functionality of this pump. Since the MexEF-OprN RND efflux pump is involved in fluoroquinolone resistance, we next assayed bacterial sensitivity against ciprofloxacin by evaluating their MICs. As shown in Table 1, both the *P. fluorescens* strains were more resistant to this antibiotic after exposure to NO_2_ than to synthetic air. Chloramphenicol is a nitroaromatic antimicrobial that is a substrate for MexEF-OprN (Köhler et al., 1997; Sobel et al., 2005). Accordingly, NO_2_-exposed *P. fluorescens* strains MFAF76a and MFN1032 were about 2 fold more resistant to this antibiotic than synthetic air-treated bacteria (Table 1). Taken together, these data suggest a possible higher activity of this efflux pump in response to NO_2_ exposure. We next followed the growth of the NO_2_-exposed *P. fluorescens* strains in DMB medium containing ciprofloxacin or chloramphenicol at the higher antibiotic concentration leading to bacterial growth (Figure 5). Data were standardized with the control, the synthetic air treated cells growth. While ciprofloxacin had no effect on NO_2_-exposed bacteria, chloramphenicol at a concentration of 25 and 100 μg/mL for strain MFAF76a and MFN1032, respectively, led to an increase in growth for the two NO_2_-exposed *P. fluorescens* strains (Figure 5). Remarkably, the statistically significant increase of bacterial growth was maintained from 2 to 10 h, suggesting a possible NO_2_ protective effect that would be conserved for 8 h after exposure. Taken together, our data show that NO_2_ induced *mexEF-oprN* gene expression, and consequently increased the resistance to ciprofloxacin and chloramphenicol.

**Table 1:** **NO_2_ exposure increases *Pseudomonas fluorescens* antibiotic resistance**.

**Strain**	**NO_2_ concentration**	**Ciprofloxacin**	**Chloramphenicol**
	**(ppm)**	**MIC (μg/mL)**	**MIC (μg/mL)**
MFAF76a	0	6.25	50
	45	12.5	>100
MFN1032	0	3.125	150
	45	6.25	200

**Figure 6:**
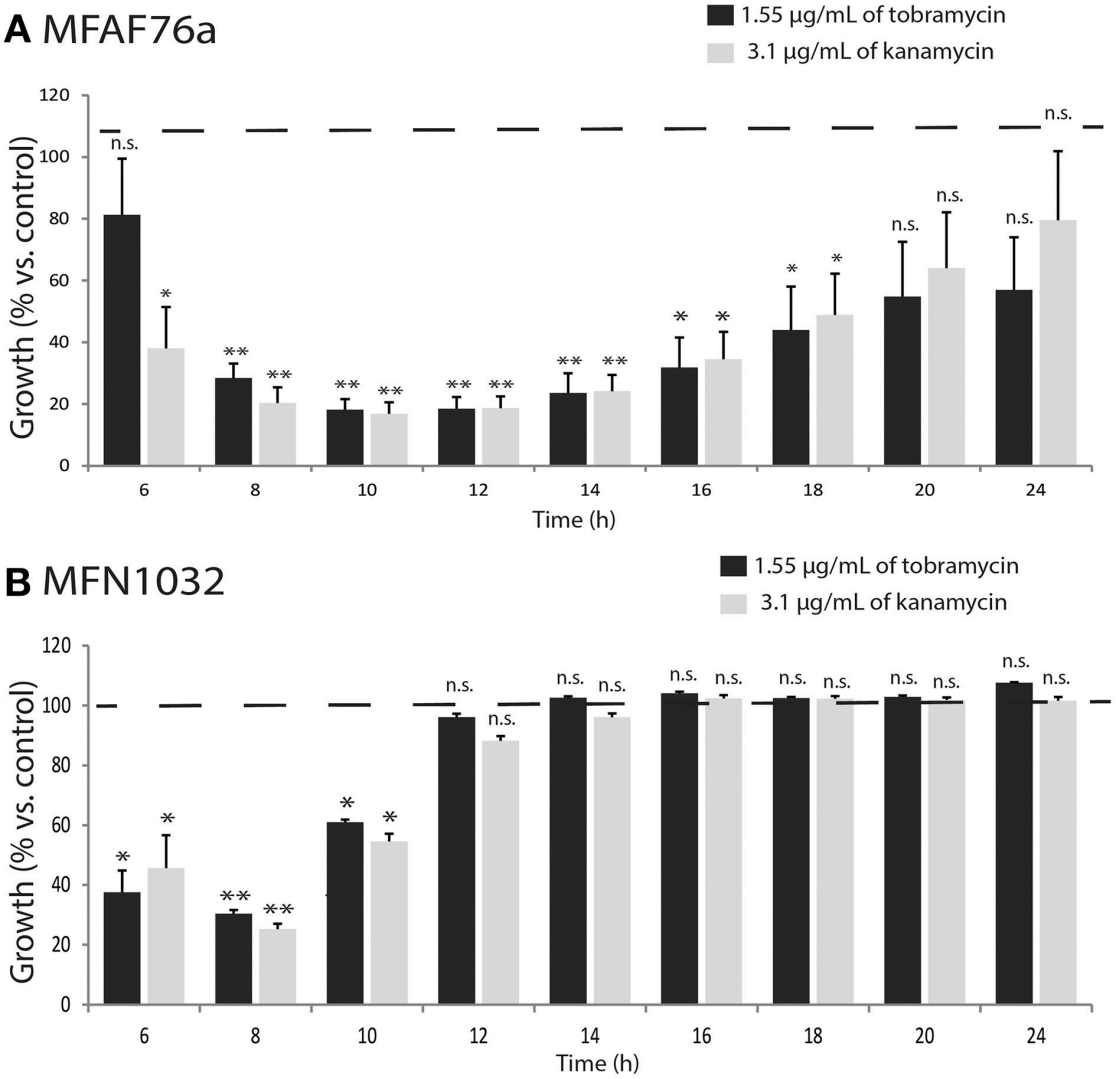
**NO_**2**_ exposure affects ***Pseudomonas fluorescens*** growth with aminoglycosides**. After 2 h exposure to 45 ppm of NO_2_, growth of airborne MFAF76a **(A)** and clinical MFN1032 **(B)** in presence of tobramycin (1.55 μg/mL; 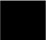) and kanamycin (3.1 μg/mL; 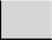) was tested. A_580_ was recorded at indicated time points. The control sample was bacteria exposed to synthetic air, and grown in presence of antibiotics in indicated concentrations. The data are presented as percentages of growth relative to air-exposed control. Pooled data from three independent experiments in duplicate ± SEM are reported. Statistical significance was calculated by the non-parametric Mann-Whitney-Test *p* < 0.05 (^*^), < 0.01 (^**^); n.s. non-significant. Dotted line shows the control (100%).

MexEF-OprN-overproducing mutants with enhanced fluoroquinolone resistance often increase bacterial susceptibility to aminoglycosides apparently owing to impairment of the MexXY system (Sobel et al., 2005; Morita et al., 2015). The effect of NO_2_ on tobramycin and kanamycin sensitivity was then assayed by performing MICs. As shown in Table 2, NO_2_ treatment led to reduce the MICs of the two tested antibiotics, suggesting that NO_2_ increases *P. fluorescens* sensitivity to aminoglycosides. Tobramycin and kanamycin, at subinhibitory concentration of 1.55 and 3.1 μg/mL respectively, were found to decrease the growth of NO_2_-exposed bacteria (Figure 6). This effect was observed only from 6 to 10 h of growth for MFN1032 and from 6 to 18 h of growth for MFAF76a, highlighting the time-limited NO_2_ effect on bacterial antibiotic sensitivity. Altogether, our data show that NO_2_ increases *P. fluorescens* sensitivity to tobramycin and kanamycin, accordingly its homolog NO is also found to increase *P. aeruginosa* sensitivity to tobramycin (Barraud et al., 2006). Noticeably, this phenotype is consistent with previously published data supporting decreasing resistance to aminoglycosides of MexEF-OprN-overproducing mutant (Sobel et al., 2005; Morita et al., 2015). Since this phenotype is often associated with the impairment of the MexXY-OprM efflux pump, we next assayed the effect of NO_2_ on the expression of the *mexXY* genes. As shown in Figure 4, NO_2_ treatment had an opposite effect on *mexXY* gene expression. While NO_2_ increased the expression of *mexXY* in the airborne strain MFAF76a, it drastically reduced production of *mexXY* mRNA in the clinical strain MFN1032. While this latter phenotype is often described in the literature (Sobel et al., 2003) as leading to increased aminoglycoside susceptibility, the overproduction of the two RND efflux pumps MexEF-OprN and MexXY-OprM is remarkable and found in very few strains, among which the multiresistant strain PA7 (Morita et al., 2015). Nevertheless, the increased expression of *mexXY* in the airborne strain cannot be related to the increased susceptibility to aminoglycosides, which we observed. Taken together, our data indicate that the NO_2_ effect on bacterial aminoglycoside resistance is complex and strain-dependent, and the up- or down- production of *mexXY* cannot account solely to explain the increased susceptibility to aminoglycosides of the two studied strains. Another hypothesis may arise related to the effects of NO_2_ on membrane properties. Indeed, the NO_2_ effect on *P. fluorescens* membrane was recently investigated, demonstrating the NO_2_-mediated modifications in both the membrane glycerophospholipids composition (i.e., ratio zwitterionic/anionic glycerophospholipids) and in the membrane electron-accepting properties (Kondakova, personal communication). It is thus conceivable that these membrane modifications would alter bacterial membrane permeability, facilitating the aminoglycoside entry into the bacterial cell.

**Table 2:** **NO_2_ decreases *Pseudomonas fluorescens* resistance to aminoglycosides**.

**Strain**	**NO_2_ concentration**	**Kanamycin MIC**	**Tobramycin MIC**
	**(ppm)**	**(μg/mL)**	**(μg/mL)**
MFAF76a	0	8.3	6.2
	45	6.2	3.1
MFN1032	0	20.0	12.5
	45	16.7	8.3

### No_2_-mediated gene expression in *p. fluorescens*

**Figure 7:**
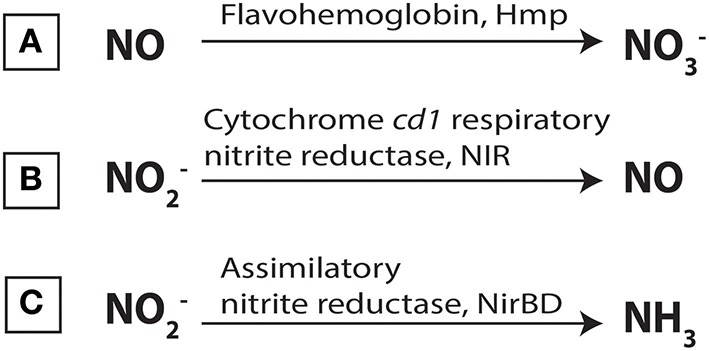
**Scheme of Hmp-mediated NO detoxification and NO_2_ reduction pathways in *Pseudomonas* spp. (A)** Flavohemoglobin (Hmp) is involved in NO detoxification acting as an NO dioxygenase to transform NO to NO${}_{3}^{-}$. The NO_2_ reduction is performed by nitrite reductase enzymes, including the respiratory cytochrome *cd1* nitrite reductase, NIR **(B)** and the assimilatory nitrite reductase NirBD **(C)**. The respiratory NIR is involved in NO${}_{2}^{-}$ reduction to NO in anaerobic conditions. NirBD takes a part of the nitrate assimilatory pathway, and reduces nitrite to ammonia.

**Figure 8:**
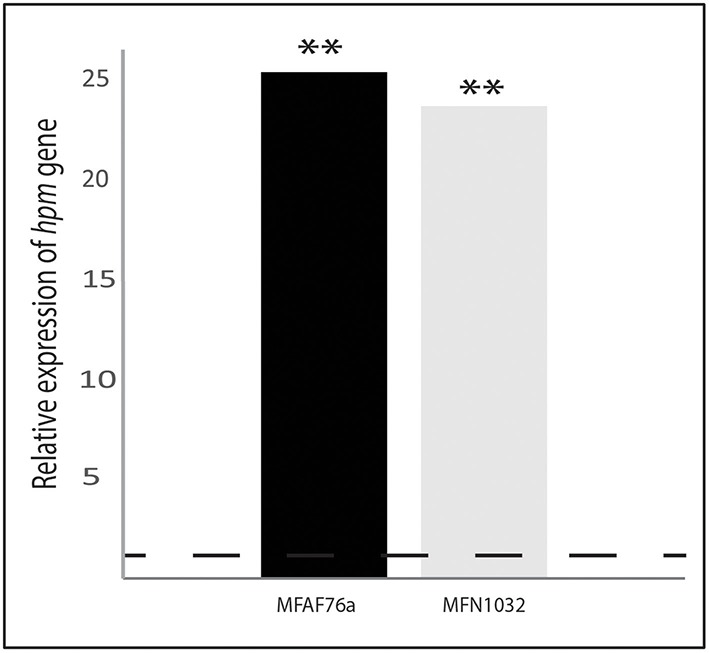
**Transcription of ***hmp*** is increased in response to NO_**2**_ exposure**. The nucleotide sequences of the *hmp*-homolog gene in *P. fluorescens* strains were obtained using the non-annotated genome drafts of airborne *P. fluorescens* MFAF76a (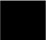) and clinical MFN1032 (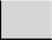). The GenBank accession numbers of *hmp* nucleotide sequences are listed in Table S2. Quantification of mRNA level was assayed using qRT-PCR on RNAs extracted from NO${}_{2}^{-}$ and synthetic air-exposed *P. fluorescens*. The PCR reactions were performed in triplicate and the standard deviations were lower than 0.15 Ct. Statistical analysis used pairwise strain comparisons (*t*-test) *p* < 0.01 (^**^). Dotted line shows the gene expression in air-exposed control.

Remarkably, we have shown herein a link between gaseous NO_2_ and soluble NO treatment. Indeed, NO is found to induce the expression of *mexEF-oprN* genes (Fetar et al., 2011) and modulates bacterial resistance to several antibiotics (Gusarov et al., 2009; McCollister et al., 2011; van Sorge et al., 2013). Since NO_2_ and NO are related chemical toxic compounds, and since NO detoxification pathways have been deeply investigated, the NO_2_ effects on several chosen target genes were tested. The most well-studied pathway for NO detoxification is based on flavohemoglobin (FlavoHb) (Hmp for *E. coli* and Fhp for *P. aeruginosa*), which acts as an NO dioxygenase to transform NO to NO${}_{3}^{-}$ (Figure 7A) (Corker and Poole, 2003; Arai et al., 2005). After exposure to 45 ppm of NO_2_, the *hmp* mRNA levels were increased almost 25- and 23-fold in MFAF76a and in MFN1032 (respectively KR818822 and KR818823 in Table S2 and Figure 8), indicating that NO_2_ induces *hmp* expression in both *P. fluorescens* and suggesting a possible involvement of Hmp in NO_2_ detoxification. The NO_2_ effect on the Hmp synthesis was observed in other studies, where, to activate the Hmp-dependent detoxification pathway, NO_2_ was proposed to be reduced to NO (Poole et al., 1996). In *Pseudomonas* spp., NO_2_ reduction can be performed by nitrite reductase (NIR) enzymes (Figures 7B,C), including the well-studied respiratory cytochrome *cd1* nitrite reductase (Figure 7B) of the denitrification pathway (Arai et al., 2005; Shiro, 2012). According to the genome draft analysis

(data not shown), both MFAF76a and MFN1032, like the majority of *P. fluorescens* strains (Redondo-Nieto et al., 2013), do not possess denitrifying genes, but harbor the genes encoding for the assimilatory nitrite reductase NirBD (Figure 7C). The latter is part of the Nas assimilatory pathway (from nitrate assimilation), where nitrate is reduced to nitrite, which is then reduced to ammonia (Jeter et al., 1984; Moreno-Vivián et al., 1999). In order to test the NO_2_ effect on the expression of *nirBD* operon, the *nirB* mRNA level (*Pfl76a_nirB* -KT186428 - and *Pfl1032_nirB* - KT070320 -, Table S2) was compared in the NO_2_-exposed or non-exposed *P. fluorescens* strains. In both strains, the mRNA level of *nirB* was not modified compared to the control condition (data not shown), indicating the absence of NO_2_ effect on the expression of genes coding for assimilatory NIR. To the best of our knowledge, the involvement of Nas pathway in NO/NO_2_ detoxification was not demonstrated. Given the presence of ammonium in DMB medium (Duclairoir-Poc et al., 2011), we think that the production of supplementary ammonium through the nitrite reduction is not appropriate. However, in order to better understand the mechanism of the NO_2_ detoxification, the Hmp-, Nir- and Nas-mediated mechanisms should be investigated in more details.

In this study, the response of airborne *P. fluorescens* MFAF76a to gaseous NO_2_, as a marker of air pollution, was for the first time investigated and compared to the response of the clinical *P. fluorescens* MFN1032 strain. We show that NO_2_ leads to increased biofilm formation through a c-di-GMP independent mechanism, reduced motility, as well as increasing ciprofloxacin, chloramphenicol resistance and aminoglycosides susceptibility. The question is now to understand how the NO_2_ leads to the observed phenotypes. NO_2_ has some similarities with it relative NO. NO_2_, like NO, induced the expression of *mexEF-oprN* genes, encoding the RND efflux pump MexEF-OprN. Its overexpression could, among others, be involved in the observed increase of *P. fluorescens* resistance to ciprofloxacin and chloramphenicol. NO_2_ induces also bacterial biofilm formation by strain-dependent mode, without c-di-GMP production variation. Thus, the high *P. fluorescens* adaptability to many environments, and a possible NO_2_ propensity to increase some bacterial antibiotic resistance and biofilm formation may diminish the effectiveness of antibiotic therapies in highly polluted area. In addition, we show the NO_2_-mediated upregulation of the *hmp*-homolog gene in *P. fluorescens*, suggesting a possible common pathway between NO and NO_2_ detoxification. Taken together, our data show that gaseous NO_2_ can be perceived by airborne bacteria, leading to physiological modifications that may be relevant for human health (biofilm formation, antibiotic resistance). In the context of the worrying increase of atmospheric NO_2_ concentrations (Bernagaud et al., 2014), these findings are of ecological relevance, especially because of the high NO_2_ concentrations, found in the close vicinity of any vehicle.

## Author contributions

TK contributed to the design of project, experiments, acquisition, analysis, interpretation of data, and wrote the manuscript. CC contributed in genes identification and qRT-PCR analysis. MB contributed to the transformation of *P. fluorescens* strains. MN and GB participated in c-di-GMP quantification. FD encouraged the study on the airborne bacteria. SC and NO participated in the design and drafted the manuscript. CDP led and coordinated the global project by conceiving the study, and participated in manuscript writing. All authors have read and approved the final manuscript.

### Conflict of interest statement

The authors declare that the research was conducted in the absence of any commercial or financial relationships that could be construed as a potential conflict of interest.
