# Supplementary figures and images for: Response to Gaseous NO2 Air Pollutant of P. fluorescens Airborne Strain MFAF76a and Clinical Strain MFN1032
Source: Front Microbiol. 2016 Mar 31;7:379. doi: 10.3389/fmicb.2016.00379 (PMC4814523; doi:10.3389/fmicb.2016.00379)

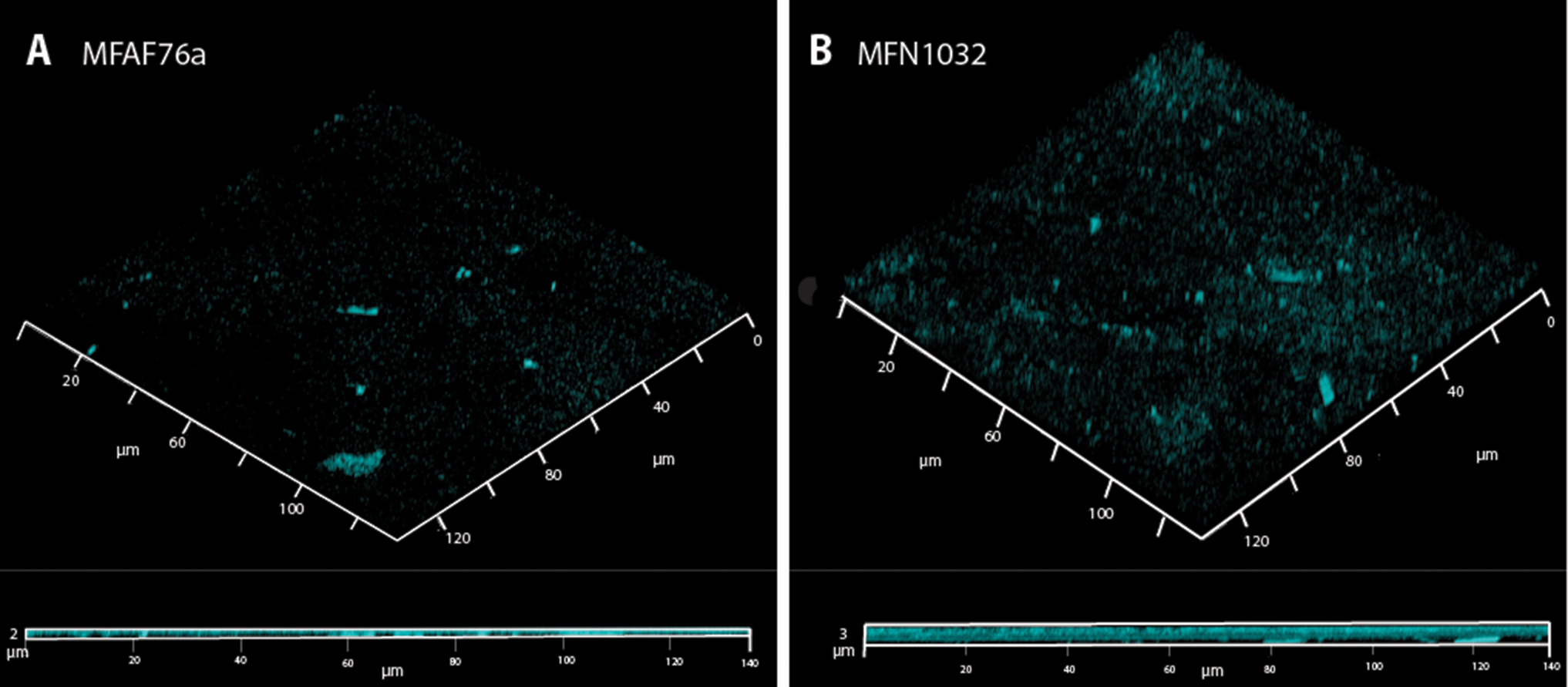

Supplement: Supplementary file 1 [file Image1.TIF]
