# Supplementary material for: Response to Gaseous NO2 Air Pollutant of P. fluorescens Airborne Strain MFAF76a and Clinical Strain MFN1032
Source: Front Microbiol. 2016 Mar 31;7:379. doi: 10.3389/fmicb.2016.00379 (PMC4814523; doi:10.3389/fmicb.2016.00379)
Supplement: Supplementary file 2 [file Table1.DOCX]

***Supplementary Material***

**Strategies implemented by airborne *Pseudomonas fluorescens* for NO_2_ detoxification. NO_2_-mediated biofilm formation and antibiotic resistance**

**Tatiana Kondakova, Chloé Catovic, Magalie Barreau, Michael Nusser, Gerald Brenner-Weiss, Marc Feuilloley, Frédéric Dionnet, Nicole Orange, Cécile Duclairoir Poc***

*** Correspondence:** Dr Cécile Duclairoir Poc: [cecile.poc@univ-rouen.fr](mailto:cecile.poc@univ-rouen.fr)

1. **Supplementary Figures and Tables**
   1. **Supplementary Figures**

**Supplementary Figure S1.** ***Pseudomonas fluorescens* population on cellulose nitrate filter before NO_2_ exposure**

Two *P. fluorescens* strains were transformed using pPSV35 plasmid harboring the CFP gene. Bacterial cultures in DMA medium, at the end of exponential growth phase were transferred on cellulose nitrate membrane filters and grown on DMA agar plates at 28°C for 4h to obtain the single layer’s bacterial population. Bacterial thickness after 4h of growth was analyzed in triplicate using confocal laser scanning microscope. Images were treated using a 3 median filter and segmented using Zen® 2009 software (Zeiss). **(A)** Representative thickness airborne *P. fluorescens* MFAF76a thickness (2 µM). **(B)** Clinical *P. fluorescens* MFN1032 thickness (3 µM).

## Supplementary Tables

**Table S1. Genes identified with the Blast 2.2.30+ software**

|  | Gene name | GenBank accession number | | Protein name | Protein description | References | |
| --- | --- | --- | --- | --- | --- | --- | --- |
|  |  | **MFAF76a** | **MFN1032** |  |  | **Strain** | **Bibliographies** |
| NO_2_ detoxification | *hmp* | KR818822 | KR818823 | flavohemoglobin | nitric oxide dioxygenase | *E. coli*  *P. aeruginosa* | Arai et al., 2005; Poole et al., 1996; Gardner et al., 1998 |
|  | *Pfl76a_nirB Pfl1032_nirB* | KT186428 | KT070320 | putative nitrite reductase large subunit NirB | assimilatory nitrite reductase | P. aeruginosa | Romeo et al., 2012 |
| Biofilm and motility | *bdlA* | KT186436 |  | putative protein BdlA | biofilm dispersion protein BdlA | P. aeruginosa | Petrova and Sauer, 2012b |
|  | *dipA* |  | KT186437 | putative phosphodiesterase DipA | protein, possessing EAL domain and involved in biofilm dispersion | P. aeruginosa | Roy et al., 2012 |
|  | *mucR* |  | KT186445 | putative phosphodiesterase MucR | protein possessing both GGDEF and EAL domains and involved in NO-mediated biofilm dispersion | P. aeruginosa | Li et al., 2013 |
|  | *ndbA* | KT186444 |  | putative phosphodiesterase NdbA | protein possessing both GGDEF and EAL domains and involved in NO-mediated biofilm dispersion | P. aeruginosa | Li et al., 2013 |
|  | *wspR* | KT186439 | KT186440 | putative diguanylate cyclase WspR | response regulator of the Wsp chemosensory system | P. fluorescens  P. aeruginosa | Newell et al., 2011; Hickman et al., 2005 |
|  | *gcbB* | KT186450 | KT186451 | putative diguanylate cyclase GcbB | diguanylate cyclase | *P. fluorescens*  *P. aeruginosa* | Newell et al., 2011; Petrova O. et al., 2014 |
|  | *gcbC* | KT186448 | KT186449 | putative diguanylate cyclase GcbC | diguanylate cyclase |  |  |
|  | *lapA* | KT186458 |  | putative large adhesive protein LapA | large adhesive protein | *P. fluorescens* | Boyd et al., 2012, 2014; Hinsa et al., 2003 |
|  | *lapD* |  | KT186447 | putative inner membrane protein LapD | inner membrane c-di-GMP effector protein |  |  |
|  | *lapG* |  | KT186446 | putative periplasmic protein LabG | periplasmic cysteine protease |  |  |
| Antibiotic resistance | *mexE* | KT070324 | KT070323 | putative membrane fusion protein MexE | RND family efflux transporter | P. aeruginosa | Maseda et al., 2000; Fetar et al., 2011; Poole K., 2005b |
|  | *mexF* | KT070321 | KT070322 | putative cation efflux protein MexF | multidrug efflux transporter |  |  |
|  | *oprN* | KT070325 | KT186432 | putative multidrug efflux RND transporter, outer membrane factor lipoprotein OprN | outer membrane component of multidrug efflux system |  |  |
|  | *mexX* | KT070313 | KT186462 | putative MexX | MexX family efflux pump subunit | P. aeruginosa | Poole, 2005a; Morita et al., 2014; Sobel et al., 2003 |
|  | *mexY* | KT070314 | KT070315 | putative MexY | multidrug efflux protein |  |  |

**Supplementary Table S2.  Primer sequences of the indicated genes used for quantitative RT-PCR assay.**

| Gene | | Forward primer (5’-3‘) | | Reverse primer (5’-3‘) | |
| --- | --- | --- | --- | --- | --- |
| *Pseudomonas fluorescens* MFAF76a | | | | | |
| *hmp* | | AAACCGCGATCTATGACCAG | | GCTTCATGCCGATGTACTGA | |
| *Pfl76a_nirB* | | AATTGAAAGTCACCGGCATC | | GAATCTGCCGGAAATACCAA | |
| *bdlA* | | ACATTCTTACCGCCAACCAG | | ACTGTCCGTGGAAGAACTCG | |
| *gcbB* | | AGTCATCTGCCGGTGAACAT | | GTCCGTCGAATTCCATTTTG | |
| *gcbC* | | CCGACAGCACACTGAACATC | | CATGCTCGAGGTACTGGTGA | |
| *lapA* | | CTCCAACACTGCGACGACTA | | TCTTGATACCGAGGCTGCTT | |
| *ndbA* | | AGATGAAAGAGCCGATCGAA | | TGAAGAAGTGCAGACCGTTG | |
| *wspR* | | TACCACTCGCGGTCCTACAT | | CGTCGATCATCAGCAATGAC | |
| *mexE* | | ACTCAAACATTTGCGCTTCC | | AATTCGTCCCACTCGTTGAC | |
| *mexF* | | GAGTGGACCGACCTGACCTA | | GCAAGTCCCACCAGTACGAT | |
| *oprN* | | ACCTCAACAACCAGCAGGAG | | AGGTCGACGGTCAGTTTGTC | |
| *mexX* | | CGCAGCGAGTTTCCCAAT | | TGGATGGTTGCCTGCTCAA | |
| *mexY* | | GGCTGGGTCGGCTATGC | | TCAGCAGGACCACGTAGATCAT | |
| *Pseudomonas fluorescens* MFN1032 | | | | |  |
| *hmp* | GATAAACCGCTGGTGCTGAT | | ATCCTCGGCGTAGCAGTAGA | |  |
| *Pfl1032_nirB* | CGCCCTATGTGCTGGTGTT | | ATCTGCCGGGCCATCTC | |  |
| *dipA* | ACGAAGACATCACCCAGACC | | TTGATGCGCTTGAAGTTGTC | |  |
| *gcbB* | CTGGCGATTTACCTGGATGT | | CTCCAGATCCAAAGGGTGAA | |  |
| *gcbC* | CGCCTGTCGTACTTTCATCA | | GCCCACATTCGAAAAACTGT | |  |
| *lapD* | TGACGAGGTTATGGGTTTCC | | ACTGGACGCGCTAAACAACT | |  |
| *lapG* | TCCCTATCGACTGCTTTGCT | | CCTGTTTTTCGCTCACCAAT | |  |
| *mucR* | CCTTGTCGTGATTTCCCTGT | | GCCAACATCCCGATAAAGTG | |  |
| *wspR* | GCTATCACTCGCGCTCCTAC | | TCATCAGCAACGAGAGTTGG | |  |
| *mexE* | CCGACAAGGTTTACGCCTAC | | GTGTATTCGCCCTTGCTGTT | |  |
| *mexF* | CTGACCCTGACCATCACCTT | | ATTGAGGATCGCGTAGTTGG | |  |
| *oprN* | GTTGCTGGCATTGGAAGAGT | | GTCGAGCAACACCAGGAAGT | |  |
| *mexX* | CCGTTGCCGGGTAAATTG | | GAACTCGCTGCGCAGGAT | |  |
| *mexY* | CAGTGGTTTTGAGTTCCGTTTG | | GCAGCGTCCAGCAATTCAGT | |  |
